# Circulating sTweak is associated with visceral adiposity and severity in patients with obstructive sleep apnea syndrome

**DOI:** 10.1038/s41598-021-01553-3

**Published:** 2021-11-11

**Authors:** Sule Cilekar, Selvihan Beysel, Savas Karatas, Aydin Balci, Kursad Akaslan, Ali Uncu

**Affiliations:** 1 Department of Chest Diseases, Faculty of Medicine, Afyonkarahisar Health Sciences University, Chest Diseases Clinic, Block C Floor: 5, Izmir Highway 3 km, 03200 Afyonkarahisar, Turkey; 2 Department of Endocrinology and Metabolism, Afyonkarahisar Health Science University, Afyonkarahisar, Turkey; 3 Department of Endocrinology and Metabolism, Istanbul Research and Education Hospital, Istanbul, Turkey; 4 Isıklar Family Health Center, Eskisehir, Turkey; 5 Eskisehir City Hospital Biochemistry Department, Eskisehir, Turkey

**Keywords:** Immunology, Biomarkers, Diseases, Health care, Health occupations, Medical research, Pathogenesis, Risk factors, Signs and symptoms

## Abstract

Hypoxia is linked to an inflammatory imbalance in obstructive sleep apnea syndrome (OSAS). Circulating soluble tumor necrosis factor (TNF)-like weak inducer of apoptosis (sTWEAK) is a cytokine that regulates inflammation and insulin resistance in adipose tissue. This study first investigated sTWEAK concentrations in patients OSAS and evaluated associations between sTWEAK concentrations and visceral adiposity, metabolic dysfunction, and hypoxia observed in OSAS. Forty age, sex, and body mass index-matched patients with simple habitual snoring (HSS) and 70 patients with OSAS were included. Patients were divided according to OSAS severity: mild-moderate (apnea–hypopnea index, AHI 5–30 events/h) and severe (AHI ≥ 30 events/h). Anthropometric data, glucose metabolism, visceral fat (VF) ratio, and sTWEAK levels were compared. sTWEAK levels were higher in the OSAS group than in the HSS group (931.23 ± 136.48 vs. 735.22 ± 102.84 ng/L, *p* = 0.001). sTWEAK levels were higher in severe OSAS than in mild-moderate OSAS (1031.83 ± 146.69 vs. 891.01 ± 110.01 ng/L, *p* = 0.002. When we evaluated the sTWEAK value and AHI, VF ratio, total cholesterol, blood pressure, homeostasis model of assessment-insulin resistance, and high-sensitivity C-reactive protein using multiple regression analysis, a significant correlation was found between sTWEAK levels and AHI (p < 0.001). It was found that sTWEAK levels were not correlated with glucose metabolism and VF ratio. Increased circulating sTWEAK levels were associated with the severity of OSAS. High sTWEAK levels were correlated with increased AHI. sTWEAK concentrations are linked to severe OSAS.

## Introduction

Obstructive sleep apnea is a common sleep-related breathing disorder. Patients with OSAS present with a clinical picture characterized by repetitive collapse of the pharynx during sleep, which causes oxygen desaturation and recurrent awakenings from sleep. Patients with OSAS may develop several cardiovascular complications such as hypertension, cardiac arrhythmias, left heart failure, coronary artery disease, right heart failure/pulmonary hypertension, and also cerebrovascular complications in the long term. The mechanisms that occur in the body secondary to hypoxemia are thought to play a role in the development of various complications. The number and severity of complications are positively correlated with the duration and degree of hypoxemia^1^.

Tumor necrosis factor (TNF)-like weak inducer of apoptosis (TWEAK) and its receptor fibroblast growth factor-inducible 14 (Fn14) is a ligand-receptor pair in the TNF superfamily^2^. Fn14 is identified as the specific TWEAK receptor. The Fn14 gene is located at the 16p13.3 chromosome, encoding a transmembrane protein. The TWEAK gene is located on the 17p13.1 chromosome, encoding a transmembrane protein that can co-express both membrane and soluble TWEAK^3^. Fn14 binds TWEAK with physiologic affinity at the extracellular domain, transducing TWEAK/Fn14 signaling^4^. Circulating soluble TWEAK (sTWEAK) is expressed in various cells such as in the intestine, tumor cell lines, liver, skeletal muscle, pancreas, and adipose tissue; Fn14 expression does not occur in healthy tissues under normal conditions^5^. After tissue damage, Fn14 expression is immediately stimulated in injured tissues, including skeletal muscle, heart, kidney, liver, and atherosclerotic vessels^2–5^.

Studies show that cell regeneration and apoptosis are regulated by increased sTWEAK values after damage and destruction due to various causes, including hypoxia in tissues. sTWEAK-induced pro-inflammatory activity is mediated through the canonic NF-KB pathway in subcutaneous adipocytes and non-canonic NF-KB pathway only in visceral adipocytes^6^. sTWEAK inhibits adipocyte differentiation and induces a moderate inflammatory state^7^. There are conflicting studies regarding that the correlation between the TWEAK/Fn14 pathway and metabolic disorders^8,9^. However, there has been no report on the concentrations of sTWEAK in visceral obesity and metabolic dysfunction in patients with OSAS. This study aimed to determine sTWEAK concentrations in patients with OSAS, and investigate the associations with metabolic disorders, visceral obesity, and hypoxemia, and potentially explain the severity of OSAS.

## Materials and methods

### Sample population

Patients who snored and/or disturbed sleep were referred to the Sleep Disorders Unit at a tertiary University Hospital between March 2017 and April 2018. The patients were investigated for symptoms of OSAS. One hundred ten consecutive subjects with suspected OSAS wereanalyzed. Forty (36.3%) patients with negative polysomnography (apnea–hypopnea index, AHI < 5, 3.65 ± 0.48) comprised the simple habitual snoring (HSS) group. 40 age, sex, and body mass index (BMI)-matched patients with HSS and 70 patients with OSAS (AHI: 20.47 ± 12.93) were included.

The exclusion criteria were as follows: patients with chronic diseases such as liver insufficiency, chronic renal failure, thyroid dysfunction, autoimmune, and/or psychiatric disorders or treated with any type of medications affecting lipid metabolism and insulin secretion. Also, patients who had a disease that might cause higher levels of sTWEAK, including myocardial infarction, chronic heart disease, chronic renal failure, and liver insufficiency were excluded.

The diagnosis of OSAS was made through cardiorespiratory sleep tests, including nasal flow, thoracic movements, body position, heart rate, and snoring. HSS and OSAS were diagnosed according to the AHI (frequency of apnea and hypopnea per hour of sleep ≥ 5/h). Overnight polysomnography (Embla Flaga Inc. Iceland) was performed for all patients in a sleep laboratory. Electroencephalography, oral-nasal airflow, electrooculography, chest and abdominal movement, pulse oximetry, and body position were recorded. A pause of airflow of more than 10 s was defined as apnea. A decrease of airflow of more than 10 s was defined as hypopnea. Oxygen desaturation means 4% or greater. AHI was assessed to investigate the severity of OSAS. OSAS was defined when AHI was ≥ 5. The patients were divided into two groups according to OSAS severity: mild-moderate OSAS (AHI 5–30 events/h) and severe OSAS (AHI ≥ 30 events/h).

All patients were assessed for anthropometric indices. Waist circumference was measured midway between the lower costal margin and iliac crest. Hip circumference was measured at the height of the greater trochanter. Based on these two values, the waist-to-hip ratio (WHR) was determined. BMI was calculated as weight (kg) divided by height (m^2^). Blood pressure (BP) was measured twice using a mercury sphygmomanometer from the right arm of patients in the sitting position after 5 min of rest, and the average value was calculated. Body composition was assessed using a two-point bioelectric impedance apparatus (BIA) calibrated for adults (VISCAN Corp.). Fasting serum glucose, insulin, triglycerides, total cholesterol (TC), high-density lipoprotein cholesterol (HDL-C), and low-density lipoprotein cholesterol (LDL-C) levels were measured. Insulin resistance (IR) was assessed using the homeostatic model assessment-insulin resistance (HOMA-IR) index equation: HOMA-IR = [fasting insulin (mIU/L) × fasting glucose (mg/dL)]/405^21^. sTWEAK levels were measured using a commercially available kit with an enzyme-linked immunosorbent assay (ELISA) (Human Tumor necrosis factor-related weak inducer of apoptosis (TWEAK) ELISA Kit, Eastbiopharm Co LTD, Hangzhou, China). The assay range was 10 ng/L and 4000 ng/L with a lower sensitivity limit of 5.53 ng/L.

This study was approved by the Afyonkarahisar Health Science University local ethics committee (2019/157), and written informed consent was obtained from all participants. All methods were performed in accordance with the relevant guidelines and regulations.

### Statistical analysis

In our study, the minimum sample size was 102, with an effect size of 0.50, 95% confidence interval, and 80% power to determine the sTWEAK concentrations of patients with OSAS, and investigate the associations with metabolic disorders, visceral obesity, hypoxemia, and potentially explain the severity of OSAS. Considering that there might be incomplete or incorrect information, measurements were made from 110 patients, and the data were evaluated.

Data were analyzed using the SPSS 20 for Windows software package (SPSS Inc., Chicago, USA). The Shapiro–Wilk test was used to determine normal distribution. Continuous variables are presented as mean ± standard deviation (SD), and categorical variables as percentages. The difference between the groups was analyzed using one-way analysis of variance (ANOVA) followed by the post hoc Tukey–Kramer test for multiple comparisons. Correlations were analyzed using Pearson and Spearman's tests. Multiple regression analysis was used to determine variables that certainly determined OSAS. *p*-values of < 0.05 were considered significant.

## Results

According to the AHI, 50 patients had mild-moderate OSAS (AHI: 13.10 ± 5.41 events/h), and 20 (71.42%) had severe OSAS (AHI: 38.90 ± 5.61 events/h) (Table 1). Age, sex, and BMI values were similar between the OSAS and HSS groups (*p* > 0.05). Insulin, HOMA-IR, and lipids were higher in the OSAS group than in the HSS group (*p* < 0.05). The visceral fat (VF) ratio (13.30 ± 3.31 vs. 10.51 ± 1.67%) was high in the OSAS group compared with the HSS group (*p* < 0.05). sTWEAK levels were high in the OSAS group compared with the HSS group (931.23 ± 136.48 vs. 735.22 ± 102.84 ng/L, p = 0.001) (Table 1).

**Table 1:** Characteristics of patients with HSS, mild-moderate OSAS and severe OSAS.

	HSS (n = 40)	Mild-moderate OSAS AHI ≥ 5 < 30 (n = 50)	Severe OSAS AHI ≥ 30 (n = 20)	*p*
Age (years)	50.68 ± 2.74	50.66 ± 10.67	53.10 ± 5.79	0.302
Female n (%)	30.28 ± 2.81	43 (86.0%)	19 (95.0%)	0.285
BMI (kg/m^2^)	33 (82.5%)^a^	30.15 ± 3.64^b^	31.89 ± 3.93^a,b^	0.579
Waist/hip ratio	0.87 ± 0.36	0.89 ± 0.05	0.91 ± 0.07	0.209
Systolic blood pressure (mmHg)	121.15 ± 8.77^a,b^	127.01 ± 6.38^a^	125.51 ± 9.01^b^	**< 0.001**
Diastolic blood pressure (mmHg)	76.85 ± 5.25	77.01 ± 5.34	76.50 ± 5.15	0.998
Triglyceride (mg/dL)	115.62 ± 35.37^a^	175.02 ± 83.21^a^	165.95 ± 109.94	**< 0.001**
Total cholesterol (mg/dL)	187.21 ± 21.99^a^	222.90 ± 40.21^a^	208.49 ± 50.42	** 0.01**
HDL-C (mg/dL)	55.22 ± 10.51^a^	48.24 ± 9.83^a,b,c^	54.20 ± 12.85^b,c^	**< 0.001**
LDL-C (mg/dL)	105.98 ± 16.24^a^	139.66 ± 33.41^a^	121.10 ± 30.81	**< 0.001**
Glucose (mg/dL)	120.55 ± 3.90	145.38 ± 36.81	131.75 ± 38.05	0.140
Insulin (µIU/mL)	8.58 ± 3.85^a^	14.03 ± 6.64^a^	12.61 ± 4.78	**< 0.001**
HOMA-IR	2.79 ± 1.86^a^	5.37 ± 3.34^a^	4.40 ± 2.58	**< 0.001**
hs-CRP (mg/dL)	2.10 ± 1.21	3.36 ± 3.27	3.12 ± 3.15	0.58
TWEAK (ng/L)	735.22 ± 102.84^a,b^	891.01 ± 110.01^a,c^	1031.83 ± 146.69^b,c^	** 0.002**
AHI	2.3 ± 2.5^a,c^	13.10 ± 5.41^a,b^	38.90 ± 5.61^b,c^	**< 0.001**
Visceral fat ratio (% body weight)	3.65 ± 0.48^a,b^	12.16 ± 2.83^a,c^	16.15 ± 2.68^b,c^	**< 0.001**

Significant values are in bold.

*HSS* Habitual simple snoring. OSAS: Obstructive sleep apnoea syndrome. BMI: Body mass index, *HDL-C* High-density lipoprotein cholesterol, *LDL-C* Low-density lipoprotein cholesterol, *HOMA-IR* Homeostasis model of assessment-insulin resistance, *LDL-C* Low-density lipoprotein cholesterol, *hs-CRP* High sensitivity C-reactive protein, *TWEAK* Tumor necrosis factor (TNF)-like weak inducer of apoptosis, *AHI* Apnea/hypopnea index.

^a^Shows the difference between which groups.

^b^Shows the difference between which groups.

^c^Shows the difference between which groups.

The VF ratio was high in the severe OSAS group compared with the mild-moderate OSAS group (16.15 ± 2.68 vs. 12.16 ± 2.83%, *p* = 0.001). Triglyceride, insulin, HOMA-IR, and hs-CRP values were similar between the severe and mild-moderate OSAS groups (p > 0.05). sTWEAK levels were high in the severe OSAS group compared with the mild-moderate OSAS group (1031.83 ± 146.69 vs. 891.01 ± 110.01 ng/L, *p* = 0.002) (Table 1).

There was a significant positive relationship between AHI and sTWEAK when we evaluated the relationship between TWEAK values and AHI, VFRT, hs-CRP, HOMA-IR, total cholesterol, and blood pressure using multiple regression tests (R^2^ = 0.419) (*p* < 0.001). No relationship was observed between sTWEAK and all other parameters after this multiple regression analysis (Table 2).

**Table 2:** Evaluation of the relationship of TWEAK variable with other parameters by multiple regression analysis.

Model TWEAK (R^2^ = 0.419)	Beta	95% confidence interval for B		*p*
		Lower bound	Upper bound	
(Constant)		418.076	2231.397	0.005
Systolic blood pres	− 0.029	− 6.408	4.655	0.754
Diastolic blood pres	− 0.109	− 12.193	2.684	0.208
Triglyceride	− 0.046	− 0.533	0.327	0.636
Total cholesterol	0.125	− 0.187	1.216	0.149
HDL-C	− 0.170	− 6.110	0.093	0.057
LDL-C	− 0.078	− 1.543	0.789	0.523
HOMA-IR	0.070	− 6.301	14.013	0.453
AHI	0.583	3.207	10.422	**< 0.01**
hs-CRP	− 0.037	− 11.525	7.302	0.657
VFRT	− 0.031	− 16.134	12.624	0.809

Significant values are in bold.

Variables included in the model: age, GDM (no/yes), family history of T2DM, previous GDM, systolic and diastolic blood pressure, HDL cholesterol, TG, prepregnancy BMI, sTWEAK.

Variables included in the model: age, GDM (no/yes), family history of T2DM, previous GDM, systolic and diastolic blood pressure, HDL cholesterol, TG, prepregnancy BMI, sTWEAK.

Variables included in the model; *TWEAK* Tumor necrosis factor (TNF)-like weak inducer of apoptosis, *HDL-C* High-density lipoprotein cholesterol, *LDL-C* Low-density lipoprotein cholesterol, *HOMA-IR* Homeostasis model of assessment-insulin resistance, *AHI* Apnea/hypopnea index, *hs-CRP* high sensitivity C-reactive protein, *VFRT* Visceral fat ratio.

## Discussion

This study showed that sTWEAK concentrations were higher in patients with severe OSAS than in patients with mild OSAS and HSS. High sTWEAK levels were positively correlated with increased AHI but not with cardiometabolic parameters and VF ratio. These findings showed that increased circulating sTWEAK concentrations were associated with the severity of OSAS.

The result of OSAS disease is hypoxemia. As the severity of OSAS increases, the depth of hypoxemia in patients also increases. Inadequate oxygen supply causes mitochondrial dysfunction and endoplasmic reticulum stress^10^. The pathophysiology of this process is complex and multifactorial. Mechanisms such as mitochondrial dysfunction, oxidative/nitrosative stress, apoptosis, necroptosis, and inflammatory processes are involved^6^. The increase in sTWEAK values due to tissue damage secondary to hypoxemia may be considered as a recovery mechanism. This situation may also be related to the inflammatory process that occurs secondary to hypoxemia. A possible mechanism is that hypoxia changes the balance between anti-inflammatory and pro-inflammatory impact on adipose tissue^6,7^. We think that it is aimed at repairing the damage that develops in the tissues. It was shown in a study that increased sTWEAK protein due to carbon monoxide poisoning caused tissue hypoxia and apoptosis^4^.

sTWEAK is a multifunctional cytokine, and it modulates several cellular processes such as the stimulation of cell proliferation, differentiation, and stimulates inflammatory cytokines, angiogenesis, inflammation, migration, and rarely apoptosis^5–7^. sTWEAK has pleiotropic effects depending on conditions^3^. In acute injury or inflammation, sTWEAK controls proliferative and inflammatory responses contributing to repair and regeneration. Nevertheless, in a chronic state characterized by persistent Fn14 up-regulation, sTWEAK can augment inflammation and induce tissue damage, fibrosis, and aberrant remodeling^2,3^. Experimental animal studies have shown that TWEAK/Fn14 signal expression is increased in tissues damaged by hypoxia^11^. When multiple regression analysis was conducted on sTWEAK values and all other parameters, especially AHI, a significant relationship was observed between sTWEAK and AHI only (Table 2). sTWEAK is associated with outcomes in OSAS. Patients with OSAS and HSS with similar BMI were included in this study to eliminate the effect of obesity on sTWEAK.

The impacts of sTWEAK on human bronchial epithelial cells in asthma and chronic obstructive pulmonary disease (COPD) have been investigated^12–15^. In patients with non-eosinophilic childhood asthma, increased levels of the expression of sTWEAK in sputum were found. It was determined that sTWEAK levels showed a positive correlation with the severity of asthma^16^. The pro-inflammatory cytokine sTWEAK has been found to contribute to chronic airway inflammation by stimulating TGF-β-induced epithelial–mesenchymal transition in human bronchial epithelial cells^12^. During the epithelial–mesenchymal transition, sTWEAK and TGF-β1 have been shown to act synergistically to induce the production of asthma-related chemokines and cytokines in human bronchial epithelial cells^13^. TWEAK/Fn14 pathway-augmented human airway smooth muscle cell proliferation and migration through activation of the canonic NF-KB signaling pathway participate in airway remodeling in asthma^14^. The TWEAK/Fn14 axis enhances human bronchial epithelial cells to increase the release of interleukin (IL)-8 and granulocyte–macrophage colony-stimulating factor (GM-CSF)^14^. sTWEAK and TNF-α cytokine expression was increased in patients with stable COPD with skeletal muscle dysfunction^17^.

sTWEAK and Fn14 are expressed in both visceral adipose tissue and subcutaneous adipose tissue^18–20^. sTWEAK is a modulator of the inflammatory/anti-inflammatory equilibrium in the insulin-resistance condition^8,9^. Inflammation can control the expression of sTWEAK in macrophages and Fn14 in adipocytes^19^. Adipocytes of patients with severe obesity had increased Fn14 concentrations in inflammatory conditions^6,19,20^. In our study, sTWEAK was not correlated with the VF ratio. The effects of sTWEAK in obesity and adipose tissue biology are conflicting. Nevertheless, exogenous sTWEAK treatment altered adipokine production and enhanced pro-inflammatory cytokines but did not disturb insulin signaling in cultured adipocytes^21^. Discordantly, it has been suggested that sTWEAK may be cardiometabolic protective.

Low sTWEAK concentrations were associated with more inadequate cardiometabolic risk in patients with type 2 diabetes and obesity, and this can be explained by the ability of sTWEAK to antagonize in vitro TNF-α activity^22^. TWEAK knock-out mice enhanced insulin signaling in muscle and liver and protected them from ectopic fat deposits and dyslipidemia. sTWEAK restricted mice's healthy adipose tissue expansion^11^. sTWEAK interfered with the differentiation of adipocytes at an early stage^23^. Nevertheless, sTWEAK did not affect the metabolic function of adipocytes including lipolysis, glucose uptake, and apoptosis^20,21^. sTWEAK/Fn14 signaling was proposed as a feature of adipocyte-macrophage crosstalk at sites of adipocyte death and subsequent maladaptive ectopic adipose tissue remodeling^22,23^. Adipocyte-derived sTWEAK has an autocrine effect on the regulation of adipose expansion and ectopic adipose tissue remodeling^20–23^.

However, clinical studies reported that decreased sTWEAK concentrations in patients with type 2 diabetes and sTWEAK concentrations were negatively associated with fasting glucose levels and visceral obesity^24^. In our study, there was no correlation between sTWEAK concentrations and glucose metabolism. Reduced sTWEAK levels were observed in patients with type 1 diabetes^25^, lower sTWEAK levels were associated with insulin resistance in gestational diabetes mellitus, and sTWEAK levels were negatively correlated with type 2 diabetes incidence and glucose metabolism-related parameters^26^. In our study, sTWEAK concentrations were not associated with cardiometabolic disturbance. Reduced sTWEAK concentrations were associated with hyperglycemia, hypertriglyceridemia, abdominal obesity, and metabolic syndrome^7^. Decreased sTWEAK levels have been negatively associated with glucose levels, HbA1c, HOMA-IR, central obesity, and a poor cardiovascular profile^20–22^. sTWEAK concentrations were negatively correlated with total cholesterol and triglycerides levels^18^. Our study showed that sTWEAK concentrations were not correlated with the lipid profile. Similarly, Yilmaz et al. reported that the atherogenic lipid profile was not associated with sTWEAK concentrations^27^. The sTWEAK release rate was low in atheroma plaques, suggesting that lipotoxicity can regulate sTWEAK concentrations^20^.

### Current limitations and future directions

The most important limitation of our study was that it could have been evaluated with a larger number of patients. The number of patients per group was low, especially when we divided them into groups according to the severity of OSAS. It only represents a limited proportion of OSA subjects.

### Conclusion

In this study, it emerged that there was a strong bond between OSAS severity and sTWEAK. As the severity of OSAS increased, the possibility of developing cardiovascular, cerebrovascular conditions related to OSAS increased. We mainly attribute the relationship between sTWEAK and OSAS to hypoxemia. We think that sTWEAK plays a crucial role in developing hypoxic adverse effects associated with OSAS and post-remodeling. This situation supports the concept that TWEAK levels can be considered a potential novel hypoxic damage biomarker with a putative protective effect of sTWEAK. Further longitudinal studies are warranted to confirm these results in different populations.

## Data Availability

The datasets used and/or analysed during the current study are available from the corresponding author on reasonable request.

## Abbreviations

AHI: Apnea/hypopnea index
BMI: Body mass index
HDL-C: High-density lipoprotein cholesterol
HOMA-IR: Homeostasis model of assessment-insulin resistance
LDL-C: Low-density lipoprotein cholesterol
OSAS: Obstructive sleep apnoea syndrome
WHR: Waist hip ratio
HSS: Habitual simple snoring
